# The Anti-Inflammatory Cytokine, Interleukin-10, Inhibits Inflammatory Mediators in Human Epithelial Cells and Mouse Macrophages Exposed to Live and UV-Inactivated *Chlamydia trachomatis*

**DOI:** 10.1155/2012/520174

**Published:** 2012-02-19

**Authors:** Abebayehu N. Yilma, Shree R. Singh, Stacie J. Fairley, Murtada A. Taha, Vida A. Dennis

**Affiliations:** ^1^Center for Nanobiotechnology and Life Science Research, Alabama State University, Montgomery, AL 36104, USA; ^2^Program in Microbiology, Department of Biological Sciences, Alabama State University, 1627 Hall Street, Montgomery, AL 36104, USA

## Abstract

*Chlamydia trachomatis* infects macrophages and epithelial cells evoking acute and chronic inflammatory conditions, which, if not controlled, may put patients at risk for major health issues such as pelvic inflammatory disease, chronic abdominal pain, and infertility. Here we hypothesized that IL-10, with anti-inflammatory properties, will inhibit inflammatory mediators that are produced by innate immune cells exposed to *C. trachomatis*. We used human epithelial (HeLa) cells and mouse J774 macrophages as target cells along with live and UV-inactivated *C. trachomatis* mouse pneumonitis (MoPn) as stimulants. Confocal microscopy employing an anti-*Chlamydia* antibody confirmed cells infectivity by day 1, which persisted up to day 3. Kinetics studies revealed that live *C. trachomatis* induced TNF, IL-6, and IL-8, as a function of time, with day-2 infection inducing the highest cytokine levels. Exogenous IL-10 inhibited TNF, IL-6, and IL-8 as secreted by day-2 infected cells. Similarly, IL-10 diminished cytokine levels as produced by macrophages exposed to UV-inactivated *Chlamydia*, suggesting the IL-10-mediated inhibition of cytokines is not restricted to live organisms. Our data imply that IL-10 is an important regulator of the initial inflammatory response to *C. trachomatis* infection and that further investigations be made into IL-10 use to combat inflammation induced by this bacterium.

## 1. Introduction

*Chlamydia trachomatis *is an obligate intracellular bacterial pathogen responsible for sexually transmitted infections worldwide [1]. For clinical purposes, *C. trachomatis* is classified into different serovars, and disease manifestations depend on the type of serovar used during infection [2]. Serovars D-K are associated with pelvic inflammatory disease, salpingitis, ectopic pregnancy, and infertility in women, and with epididymitis and proctitis in men. Strains of Lymphogranuloma venereum (LGV, serovars L1–L3) cause more systemic infections that result in genital ulcers, inguinal lymphadenopathy, and acute proctitis in men. Furthermore, serovars L1–L3 are known in manifesting chronic inflammatory diseases [1–3].

*Chlamydiae* have a unique developmental cycle that begins with attachment of infectious but metabolically inactive elementary bodies (EB) to host cells surfaces. The infectious particles of *C. trachomatis* invade the mucosal surface of the female genital tract and persist in them for a long time [4]. Like other infectious organisms, *Chlamydia* infection of epithelial cells mucosal surface evokes proinflammatory cytokines such as interleukin (IL)-6, IL-8, and tumor necrosis factor (TNF) [5]. IL-8 recruits neutrophils to phagocytose the antigen that triggers pattern recognition receptors such as Toll-Like Receptors (TLR) to stimulate repair responses [6]. Excessive production of IL-6, TNF, and IL-8 contributes to disease manifestation by damaging neighboring cells as demonstrated by various investigators [7, 8]. For instance, continuous IL-8 production promotes the infiltration of neutrophils that are not only inefficient in resolving the infections but can also release protease that damages cells [9, 10]. These findings imply the importance of controlling inflammation during disease manifestation.

IL-10, an anti-inflammatory cytokine, is secreted under different conditions of immune activation by a variety of cell types, including T cells, B cells, and monocytes/macrophages [11–13]. Although IL-10 is classified as a Th2-type cytokine, it has been shown to suppress a broad range of inflammatory responses and is known to be an important factor in maintaining homeostasis of overall immune responses [14, 15]. Thus, novel therapies using IL-10 have been developed for several human diseases such as allergic responses and autoimmune diseases [16, 17]. Little is known about the anti-inflammatory effect of IL-10 during a *C. trachomatis* infection.

In this study, we explored the hypothesis that IL-10, with anti-inflammatory properties, will inhibit inflammatory mediators that are produced by innate immune cells after their exposure to *C. trachomatis.* To address this hypothesis, we used human epithelial (HeLa) cells and mouse J774 macrophages as target cells, along with live and UV-inactivated *C. trachomatis* mouse pneumonitis (MoPn) as stimulants. We first verified that J774 macrophages and HeLa cells could be infected by* C. trachomatis *mouse pneumonitis (MoPn) in our *in vitro* model system. Then we performed dose and kinetic experiments on both cell lines to establish the optimum conditions for the production of IL-8, IL-6, and TNF inflammatory cytokines, in response to live *C. trachomatis* infection. After optimization conditions were established, we investigated the ability of human or mouse recombinant IL-10 to regulate the expression of these cytokines as induced by live *C. trachomatis*. Lastly, we determined the effectiveness of IL-10 regulation of IL-6 and TNF as induced in mouse J774 macrophages exposed to UV-inactivated *C. trachomatis *as compared to that of live* C. trachomatis*. Herein, we present our results and discuss the potential role of IL-10 in regulating cytokine production levels during the early stage of a *C. trachomatis* infection.

## 2. Materials and Methods

### 2.1. Cell Lines and Culture

 HeLa cells (CCL-2) and mouse J774 macrophages were obtained from the American Type Culture Collection (ATCC, Manassas, VA). HeLa cells were cultured in minimal essential medium (MEM/H) (Sigma, St Louis, MO, USA) supplemented with 10% heat-inactivated fetal bovine serum (FBS) (Gibco, Invitrogen, Carlsbad, CA), 2 mM L-glutamine (Invitrogen) and 1 *μ*g/mL antibiotic and antimycotic (Invitrogen). Mouse J774 macrophages were cultured in Dulbecco Modified Eagle Medium (DMEM) (ATCC) supplemented with 10% heat-inactivated FBS, 1 *μ*g/mL antibiotic and antimycotic (Invitrogen). All cells were maintained at 37°C in a humidified incubator containing 5% CO_2_ for various periods of time, depending on the experimental procedure. Live *C. trachomatis* was incubated with cells in antibiotic-free medium. All cultures were subsequently centrifuged at 450 ×g at 4°C for 10 min to collect cell-free supernatants. Supernatants were stored at −80°C until they were used.

### 2.2. Infectivity

*C. trachomatis* MoPn Nigg II was purchased from ATCC (ATCC #VR-123) and propagated in HeLa cell monolayers in MEM/H supplemented with 10% FBS. The resulting infectious particle (EBs) was purified by ultracentrifugation on sodium diatrizoate (Sigma). Purified *C. trachomatis* EBs were suspended in Sucrose-Phosphate Glutamic acid (SPG) buffer, aliquoted, and stored at −80°C until used. UV-inactivated *C. trachomatis* was obtained by exposing EBs to Handle UV lamp, LW/SW, 6 W (Model: UVGL-58, Cat no. G-1605 Science Company, Denver, CO) for 3 hr at a distance of 5 cm. The desired IFU for both live and UV-inactivated EBs used in this study were calculated from the original *C. trachomatis* EB-purified stock.

To establish infection, cells (HeLa or J774) were plated at 10^5^/mL/well in 24-well plates for 24 hr after which they were infected with various concentrations of *C. trachomatis* infectious particles in 500 *μ*L of growth media/well. To assure successful penetration, target cells were infected with live organisms in the presence of 1 *μ*g/mL of cycloheximide. The cells were then incubated at 37°C under 5% CO_2_ for three different infection time-points (days 1 to 3). At each infection time point, when the infection was completed, the growth media were discarded and replaced with fresh medium in the absence of cyclohexamide. From infected HeLa and J774 cells, cumulative culture supernatants were collected every 24 hr without replacing with fresh media. On the other hand, from infected HeLa cells, noncumulative supernatants were collected every 24 hr by replacing with fresh media every time the supernatants were removed. In separate experiments, J774 cells were stimulated with various concentrations (10^3^, 10^4^, 10^5^, and 10^6^ IFU) of UV-inactivated *C. trachomatis* for 24 hr. As positive controls, HeLa (10^6^ cells/well) and J774 (10^6^ cell/well) cells were stimulated with *E. coli* LPS (1 *μ*g/mL), and culture supernatants were collected at 24, 48, and 72 h after stimulation. Collected supernatants were centrifuged at 450 ×g for 10 min at 4°C and stored at −80°C until used. Infectivity of cells with *C. trachomatis* was confirmed by immunofluorescence as described below under confocal microscopy.

The effect of recombinant IL-10 (rIL-10) on the production of TNF, IL-6, and IL-8 was evaluated using 2-day infected cells since this infection time-point induced the highest levels of cytokines. HeLa or J774 cells were infected with *C. trachomatis *(10^4^ IFU/well) for two days after which the media were replaced with media containing various concentrations (0.1, 1, 10, 100 ng/mL) of human or mouse rIL-10 (BD Biosciences, San Jose, CA, USA). Human rIL-10 was used for HeLa cells and mouse rIL-10 for J774 macrophages studies. Cell-free supernatants were collected after an additional 24 hr following centrifugation at 450 ×g for 10 min at 4°C and stored at −80°C until used. In separate experiments, J774 cells were stimulated for 24 hr with UV-inactivated *C. trachomatis* (10^6^ IFU) in the presence or absence of 10 ng/mL of rIL-10. The concentrations of UV-inactivated *C. trachomatis* and rIL-10 used for these studies were determined from dose-response curve experiments. The effect of IL-10 on cytokines as induced by LPS in HeLa and J774 cells was also evaluated to serve as a positive control.

### 2.3. Cytokines

All reagents and antibodies for cytokine ELISAs were purchased from BD Biosciences, and ELISAs were performed according to the manufacturer suggested protocol. Absorbance was read at 450 nm using a microplate reader (Chameleon, IN/USA Systems).

### 2.4. Confocal

To determine *C. trachomatis* infectivity of cells, HeLa or J774 cells (2.5 × 10^4^ cells/well) were cultured on sterilized 8-well chamber slides for 24 hr prior to addition of *C. trachomatis* (2.5 × 10^3^ IFU/well). After 1, 2, and 3 days after infection, the supernatants were removed and the cells were washed, fixed with isotonic 2% paraformaldehyde (PFA), and subsequently subjected to immunostaining using an antichlamydial antibody (Meridian Life Science, Cincinnati, OH) and FITC-labeled secondary antibody (Invitrogen) diluted in 10% normal goat serum. After 1 hr incubation at room temperature, the slides were counterstained with DAPI combined with antifade mounting solution (Invitrogen). *Chlamydia* was visualized by using a Nikon Eclipse Ti Confocal Microscope (Nikon Instrument, Melville, NY).

### 2.5. Statistics Analysis

All the data are expressed as mean ± SD of samples run in triplicate from two to three different experiments. Data were analyzed by using the two-tailed unpaired Student's *t*-test. *P* < 0.005 was considered significant.

## 3. Results

### 3.1. Infection of HeLa and J774 Cells with *C. trachomatis*

**Figure 1:**
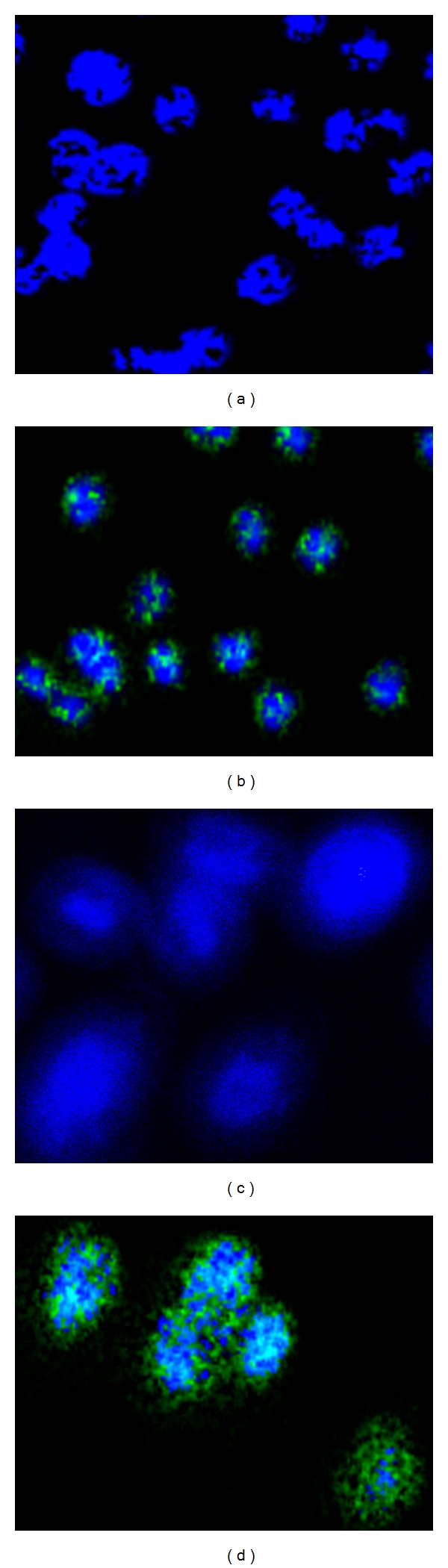
Photomicrographs showing infectivity of cells with* C. trachomatis *. Mouse J774 macrophages (a and b) and HeLa (c and d) (2.5 × 10^4^ cells/well) were infected with *C. trachomatis* EBs (2.5 × 10^3^ IFU/well) and incubated for 2 days at 37°C and 5% CO_2_. Harvested cells were stained with a monoclonal antibody to *C. trachomatis* and an FITC-labeled secondary antibody (green) and the nuclei counterstained with DAPI (blue). *Chlamydia* was visualized by confocal fluorescence microscopy.

**Figure 2:**
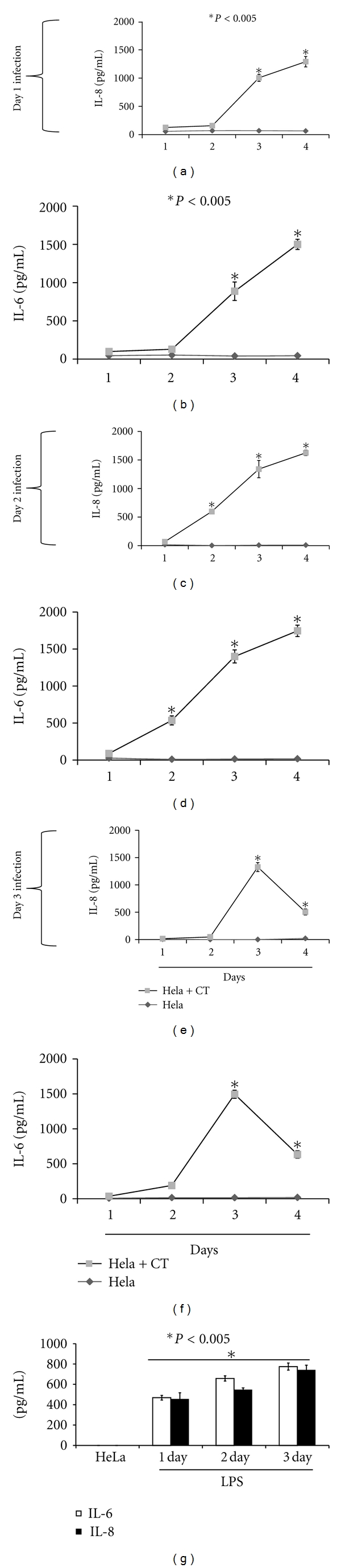
Expression profiles of IL-8 and IL-6 produced in *C. trachomatis-*(CT-) infected HeLa cells cumulative supernatants. HeLa cells (10^5^ cells/mL/well) were seeded in 24-well plates. Twenty-four later cells were infected with *C. trachomatis* EBs (10^4^ IFU/well) and after day 1 (a and b), day 2 (c and d), and day 3 (e and f) infection of cells, supernatants were collected daily without replacing with fresh media (cumulative). Human IL-8 and IL-6 cytokines were measured using cytokine-specific ELISAs. As a positive control HeLa cells (10^6^ cell/mL/well) were stimulated with LPS (1 *μ*g/mL) for various time-points (g). Asterisk indicates significant difference (*P* < 0.005), and *P* values were calculated by use of Student's* t-*test. Each bar represents the mean ± SD of samples run in triplicates. The data are representative of two separate experiments.

We first examined the infectivity of *C. trachomatis* in HeLa and J774 cells using an antichlamydial antibody and confocal microscopy. As compared to uninfected HeLa and J774 cells (Figures 1(a) and 1(c)), 2-day infected cells revealed aggregation of green fluorescence around the nucleus, which indicates infectivity of *C. trachomatis* in the cell cytoplasm (Figures 1(b) and 1(d)). Thus, the 2-day infection time-point was chosen to test the anti-inflammatory effect of IL-10 on the production levels of cytokines as induced by *C. trachomatis-*infected HeLa and J774 cells. When isotype control antibodies were used, no green fluorescence was observed (data not shown). 

### 3.2. Quantification and Kinetics of IL-6 and IL-8 in *C. trachomatis*-Infected HeLa Cells

Because accumulation of cytokines induced by *C. trachomatis* target cells are known to play key roles in inflammation, we compared their levels in cumulative culture supernatants collected from day-1 to-3 infected cells, as a function of time 1 to 4 days after infection. The kinetics of IL-8 and IL-6 production in cumulative cultured supernatants are depicted in Figures 2(a)–2(f), where their concentrations increased in a statistically significant (*P* < 0.005) manner as compared to that of uninfected cumulative supernatants. Detection of IL-8 and IL-6 in day-1 and-3 infected cell culture supernatants begin at 2 days after infection (Figures 2(a) and 2(b), 2(e) and 2(f)). In contrast, IL-8 and IL-6 cytokines were detected as early as 1 day after infection in day-2 infected cell-culture supernatants (Figures 2(c) and 2(d)), suggesting the induction of cellular signaling pathways that lead to a rapid increase in the expression of these mediators. 

 Both IL-6 and IL-8 levels gradually increased up to 4 days after infection in day-1 and-2 infected cell culture supernatants, suggesting additive secretion of these cytokines over time (Figures 2(a)–2(d)). This was not observed in day 3 infection cultures where their levels declined by 4 days after infection. LPS-stimulated cells which served as positive control similarly produced significant (*P* < 0.005) IL-6 and IL-8 cytokines when compared with that of unstimulated cells (Figure 2(g)). 

**Figure 3:**
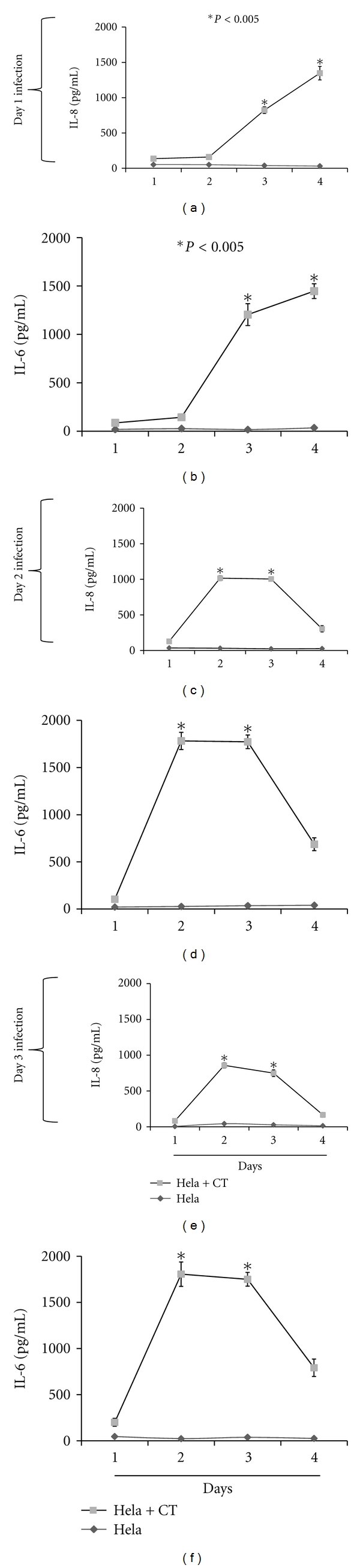
Expression profiles of IL-8 and IL-6 produced in *C. trachomatis-*(CT-) infected HeLa cells noncumulative supernatants. HeLa cells (10^5^ cells/mL/well) were seeded in 24-well plates. Twenty-four hours later cells were infected with *C. trachomatis* EBs (10^4^ IFU/well) and after day 1 (a and b), day 2 (c and d), and day 3 (e and f) infection, cell-free culture supernatants were collected daily and replaced with fresh media (noncumulative). Human IL-8 and IL-6 cytokines were measured using cytokine-specific ELISA. Asterisk indicates significant difference (*P* < 0.005), and *P* values were calculated by use of Student's* t-*test. Each bar represents the mean ± SD of samples run in triplicates. The data are representative of two separate experiments.

Since cytokine production pattern is highly influenced by the presence of growth factors that are secreted by cells, we additionally measured IL-6 and IL-8 in noncumulative culture supernatants collected from day-1 to-3 infected cells as a function of time 1 to 4 days after infection. In day-1 infected cell cultures the levels of IL-6 and IL-8 steadily increased with time (Figure 3(a) and 3(b)) as compared to day-2 and-3 infected cell cultures, where their concentrations drastically decreased by 4 days after infection (Figures 3(c)–3(f)). The production patterns of IL-6 and IL-8 in day-1 noncumulative infected cell cultures (Figures 3(a) and 3(b)) resembled that observed for cumulative day-1 infected cultures (Figures 2(a) and 2(b)). However, their production patterns were different for day-2 and-3 infected cell cultures as their levels declined remarkably by 4 days after infection (Figures 3(c) and 3(f)). The differences in the secretion patterns of IL-8 and IL-6 in cumulative and noncumulative infected cell-culture supernatants may be attributed to the fact that cytokines levels are dependent on the presence of growth factors, which are highly needed for signaling processes to regulate their production. We also measured the production of TNF in both scenarios but the values were below the detection limit. Overall our results show the induction of IL-6 and IL-8 in HeLa cells exposed to live *C. trachomatis* and that the levels of these cytokines were elevated for a prolonged period of time.

### 3.3. Dose-Dependent Requirement for IL-6 and TNF Production in *C. trachomatis*-Infected Mouse J774 Macrophages

**Figure 4:**
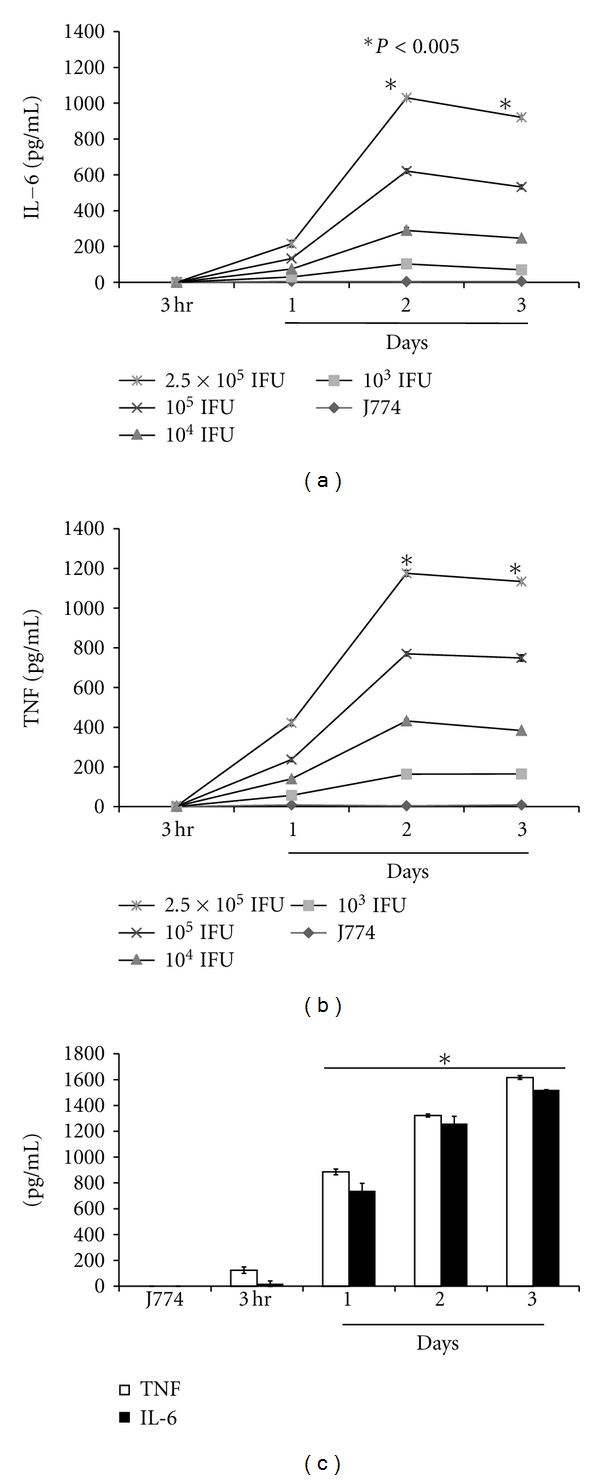
The production of IL-6 and TNF were induced in a time-dependent fashion in *C. trachomatis-*(CT-) infected mouse J774 macrophages. J774 cells (10^5^ cells/mL/well) were seeded in 24-well plates. Twenty-four hours later, cells were infected with *C. trachomatis* EBs (10^4^ IFU/well) and cell-free cultured supernatants were collected at different time-points. The levels of IL-6 (a) and TNF (b) were measured in supernatants by cytokine-specific ELISA. For positive control, J774 cells (10^6^ cell/mL) were stimulated with LPS (1 *μ*g/mL) (c) for various time-points. Asterisk indicates significant difference (*P* < 0.005), and *P* values were calculated by use of Student's* t-*test. Each bar represents the mean ± SD of samples run in duplicates. The data are representative of two separate experiments.

Because there is a complex interaction between macrophage and *C. trachomatis* as it is known to infect these cells [18], here we tested their interactions by measuring the accumulation of IL-6 and TNF in day-2 infected cell culture supernatants, as a function of time at 3 hr and at 1–3 days after infection. Our results show significant (*P* < 0.005) accumulation of IL-6 (Figure 4(a)) and TNF (Figure 4(b)) in *C. trachomatis-*infected J774 cells as compared to that of uninfected cells. The accumulation of IL-6 and TNF commenced as early as 1 day after infection with their concentrations increasing with increasing concentrations of *C. trachomatis. *Maximum IL-6 and TNF levels were observed at 2 days after infection and remained steady thereafter up to 4 days after infection (Figures 4(a) and 4(b)), suggesting a complex interaction exists between *C. trachomatis *and macrophages. We also measured the production of IL-6 and TNF in LPS-stimulated J774 cells as a positive control and observed similar trends as those seen in infected cells where a steady increase in IL-6 and TNF was observed (Figure 3(c)). Our results show the ability of *C. trachomatis* to elicit the production of IL-6 and TNF in J774 cells and overall revealed that the levels of both cytokines remained significantly elevated with the persistence of *C. trachomatis* in cells.

### 3.4. The Effect of IL-10 on the Levels of IL-6, IL-8, and TNF Induced by *C. trachomatis*-Infected HeLa and J774 Cells

IL-10 is known to downregulate the production of several inflammatory mediators as induced by a variety of innate immune cells including epithelial cells and macrophages [12]. Therefore, we examined whether IL-10 would affect the concentration levels of IL-6, IL-8, and TNF as secreted from *C. trachomatis*-infected HeLa or J774 cells. Since there is a possibility that infected HeLa and J774 cells may secrete IL-10, we first sought to measure endogenously produced IL-10. Surprisingly, IL-10 was below the detection limit of the ELISA, suggesting either the inability of *C. trachomatis* to elicit the production of this cytokine or that it may be tightly regulated by the bacteria or other mediators in the milieu.

**Figure 5:**
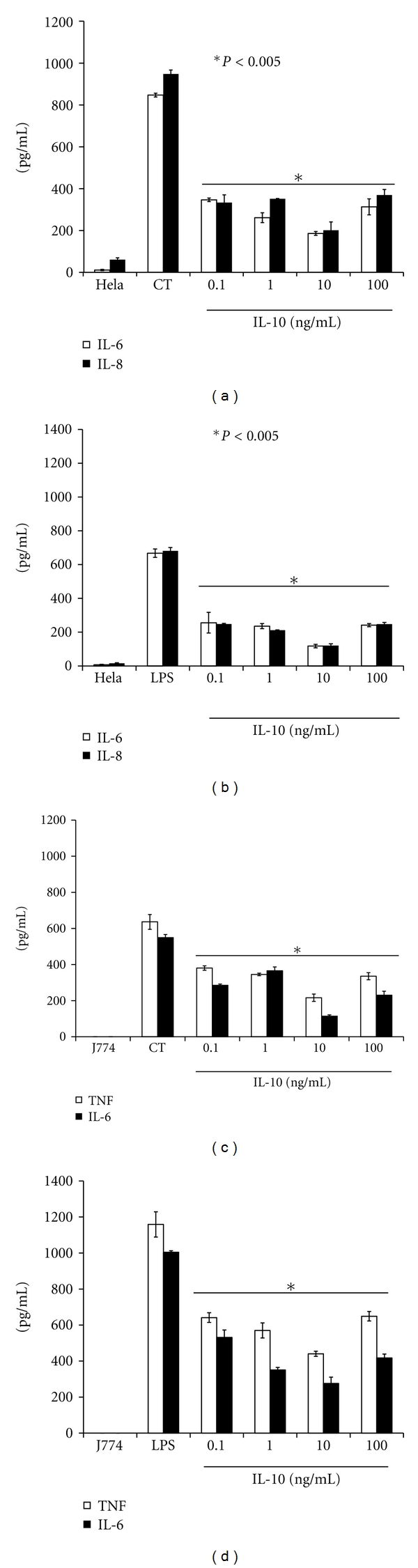
Recombinant IL-10 downregulates the production of IL-6, IL-8, and TNF in *C. trachomatis-*(CT-) infected J774 and HeLa cells. HeLa or J774 cells (10^5^ cells/mL/well) were seeded in 24-well plates and 24 hr later, cells were infected with *C. trachomatis* EBs (10^4^ IFU/well). After 2 days infection, various concentrations of recombinant IL-10 were added to cell cultures and supernatants were collected 1 day later to quantify the levels of human IL-6 and IL-8 (a) and mouse IL-6 and TNF (b) by ELISA. For positive control, HeLa and J774 cells were stimulated for 24 hr with LPS in the presence of various concentrations of IL-10, and human IL-8 and IL-6 (b) and mouse IL-6 and TNF (d) were determined from collected supernatants by ELISA. Asterisk indicates significant difference (*P* < 0.005) and *P* values were calculated by use of Student's* t-*test. Each bar represents the mean ± SD of samples run in duplicates. The data are representative of two separate experiments.

We next investigated whether or not various concentrations of exogenous IL-10 (0.1 to 100 ng/mL) added to day-2 infected HeLa or J774 cells was able to affect the production levels of IL-6, IL-8, and TNF measured at 2 days after exposure of IL-10 to infected cells. This time-point was chosen because maximum cytokines were produced at 2 days after infection in both HeLa-and J774-infected cells. The production levels of IL-6 and IL-8 were significantly reduced (*P* < 0.005) (Figure 5(a)) in supernatants of infected HeLa cells with as little as 0.1 ng of added IL-10 per mL. Similarly, the levels of IL-6 and TNF were significantly reduced (*P* < 0.005) (Figure 5(b)) in supernatants of *C. trachomatis*-infected J774 cells. In parallel with our *C. trachomatis* experiments, we measured the effect of IL-10 on LPS-stimulated production of cytokines in J774 and HeLa cells. The presence of IL-10 diminished the production levels of IL-6 and IL-8 in LPS-stimulated HeLa cells (Figure 5(b)). Similarly, the production levels of IL-6 and TNF diminished in the presence of IL-10 in LPS-stimulated J774 cells (Figure 5(d)). Overall, our results indicate that exogenously added IL-10 reduced the levels of IL-6, IL-8, and TNF in *C. trachomatis-*infected epithelial cells and macrophages and provides evidence for an anti-inflammatory role of IL-10 during an early *C. trachomatis* infection.

### 3.5. IL-10 Inhibits the Production of TNF and IL-6 Induced by J774 Cells Exposed to UV-Inactivated *C. trachomatis*

**Figure 6:**
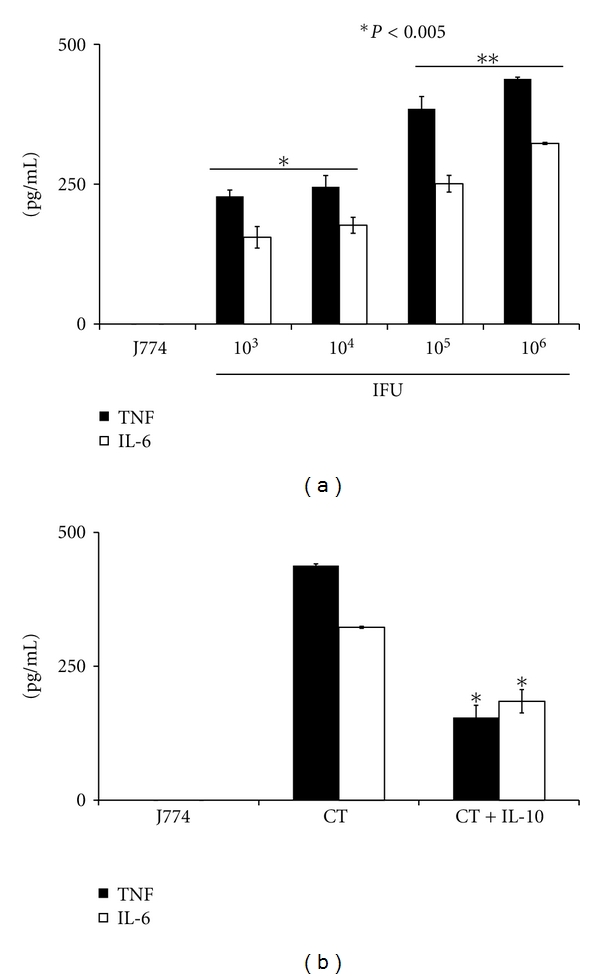
UV-inactivated *C. trachomatis* induction of TNF and IL-6 levels in mouse J774 macrophages are downregulated by exogenous IL-10. J774 cells (10^5^ cells/well) were stimulated for 24 hr with various concentrations of UV-inactivated *C. trachomatis*. The production of TNF and IL-6 was quantified in cell-free culture supernatants using cytokine-especific ELISAs. Shown are the dose-dependent TNF and IL-6 responses (a) and the downregulatory effect of IL-10 on these cytokines (b). Asterisk indicates significant difference (*P* < 0.005), and *P* values were calculated by use of Student's* t*-test. Each bar represents the mean ± SD of samples run in triplicates. The data are representative of two separate experiments.

Recently, Yu and colleagues [19] showed differences in cytokine secretion patterns in cell stimulated with live and dead *C. muridarum.* Given that cytokines induced by dead organisms may contribute to the inflammatory process, we next compared TNF and IL-6 production levels by macrophages exposed to UV-inactivated *C. trachomatis* to that exposed to live *C. trachomatis.* J774 cells stimulated with UV-inactivated *C. trachomatis* produced significantly (*P* < 0.005) more TNF and IL-6 in a dose-dependent manner as compared with unstimulated cells (Figure 6(a)). However, the levels of these cytokines (even at 10^6^ IFU) were less than those induced by live *C. trachomatis* (Figures 4 and 5), suggesting differences in the ability of live and UV-inactivated *C. trachomatis* to elicit the production of these cytokines in J774 cells. We next investigated whether or not IL-10 also dampens the levels of UV-inactivated *C. trachomatis-*induced cytokines. As shown in Figure 6(b), the production levels of IL-6 and TNF elicited by UV-inactivated *C. trachomatis* were significantly inhibited (*P* < 0.005) in the presence of added IL-10 at 10 ng/mL. This result clearly demonstrates that the IL-10 anti-inflammatory effect is not limited only to cytokines induced by live *C. trachomatis* but also to that induced by UV-inactivated *C. trachomatis.*

## 4. Discussion

Anti-inflammatory mediators, especially IL-10 has been shown to play greater roles in counterbalancing the proinflammatory response in various infectious diseases [16, 17, 20]. To date, there is no information that demonstrates the downregulatory role of IL-10 in *C. trachomatis* infection. Therefore, here for the first time we demonstrated the ability of IL-10 to downregulate the release of proinflammatory mediators evoked by *C. trachomatis* in human epithelial cells and mouse macrophages. The significance of these findings is discussed in the context of IL-10 potential role in reducing inflammation during an early *C. trachomatis* infection. We viewed this scenario as a model of the initial phase of infection, when acquired immunity has not yet developed and when the contributions of lymphocytes are almost none.

In the present study, analysis from cumulative supernatants revealed increases of IL-6 and IL-8 production after 2 and 3 days infection of HeLa cells with *C. trachomatis*, which were decreased over time in noncumulative supernatants. The higher increase of cytokines in cumulative supernatants is most likely indicative of the additive secretion of *C. trachomatis-*inducible cytokines. Alternatively, this also may be due to the fact that *Chlamydia* takes between 48 to 72 hr to complete its life cycle [21], where at the end it ruptures from cells and invades other cells subsequently inducing heightened inflammatory responses. On the other hand, interruption of this cycle may have resulted in lower production of cytokines in noncumulative supernatants. Another plausible explanation for lower levels of cytokines in noncumulative supernatants could be stress-induced stimuli resulting from removal of growth factors that are needed to sustain and maintain cell proliferation and thus enhanced secretion of cytokines. Indeed stress conditions provide different environments for cells to exhibit various cytokine expression profiles. For instance, Mittal et al., 2009 [22], demonstrated the role of iron in the production of IL-8 in *C. trachomatis-*infected HeLa cells, where the presence of iron greatly enhanced IL-8 production.

Despite the above differences between cumulative and noncumulative supernatants, after 24 hr postinfection, IL-6 and IL-8 levels were increased, suggesting that the interaction of *C. trachomatis *with HeLa cells allowed a signal that sustained intensive IL-6 and IL-8 expression. As the infection progressed, *C. trachomatis *multiplied to form inclusion bodies, subsequently stimulating excessive production of IL-6 and IL-8 as clearly shown in infected J774 cells. Several investigators have reported the production of inflammatory mediators by HeLa cells after* Chlamydial* infection using different serovars [2, 22], but the present study, to the best of our knowledge, is the first to show the ability of *C. trachomatis* MoPn Nigg II to induce cytokines in mouse J774 macrophages and human epithelial cells.

It is well known that proinflammatory cytokines play an important role in* Chlamydial* immunopathology. Multiple studies have shown the need of IL-6 and IL-8 for an early optimal host response against *Chlamydia* infection [5, 6]. However, overproduction of IL-6 and IL-8 may be toxic and damage neighboring cells. Continuous production of IL-8 promotes, for instance, infiltration of neutrophils that are not only inefficient in resolving *Chlamydial* infections but can also release protease that damage cells [7]. Therefore, our approach to reduce the production of IL-6, IL-8, and TNF during *C. trachomatis* infection can serve as an alternative way to control excess inflammation exerted from *C. trachomatis.*

It has been reported that endogenously produced IL-10 differentially regulates chlamydial infections among inbred mouse strains [23]. For example, using the murine model of *C. trachomatis *MoPn lung infection, IL-10 inhibits host clearance of chlamydial infection in the susceptible BALB/C mouse strain (which produces more IL-10) and negatively regulates inflammatory responses in the resistant C57BL/6 mouse (which produces less IL-10) [23]. Using primed spleen cells, Yang et al. [20] have shown further that the levels of IFN-*γ*, TNF, and IL-12 were decreased when exogenous IL-10 was added to *Chlamydia-*infected C57BL/6 IL-10 knockout mice, indicating that IL-10 reduces the levels of cytokines produced during the infection process in the lung model. These data suggest that IL-10 plays a crucial role in host clearance of a *Chlamydia* infection as well as regulating its induced inflammation. Although we did not observe detectable levels of IL-10 from our *C. trachomatis-*infected cells in this paper, our results clearly demonstrated that exogenous IL-10 decreased the levels of TNF, IL-6, and IL-8 in a dose-dependent manner in *C. trachomatis-*infected HeLa and J774 cells (Figure 5). This interesting finding strongly provides a plausible significance of IL-10 specifically in curtailing an excessive inflammatory response induced by *C. trachomatis *during the early phase of infection before the development of adaptive immunity.

We show in the present study that IL-10 inhibits UV-inactivated *C. trachomatis*-induced cytokine responses in innate immune cells. These cytokine responses, however, were lower as compared to that induced by live organisms. As a survival mechanism, live *C. trachomatis* exists in two morphological states (EB and RB) [4]. At the RB state, *C. trachomatis *will divide and increase its pathogen load and under this event high levels of proinflammatory cytokines are expected to be produced. However, when EBs are inactivated the conversion from EB to RB does not occur and the pathogen load remains in a steady state; therefore, reducing proinflammatory cytokines secretion levels. Here both scenarios were observed (Figures 4 and 6) where responses to live EBs were significantly higher than those to UV-inactivated EBs. Alternatively, differences in the type of antigens presented to macrophages by live and UV-inactivated *C. trachomatis* potentially may activate different pathways or receptors for inducing TNF and IL-6. Current studies are ongoing to investigate the mechanism(s) by which IL-10 regulates cytokine production evoked by live and UV-inactivated *C. trachomatis *in innate immune cells. Our result now add *C. trachomatis* to the list of other bacteria pathogens, whose cytokines production levels are altered by IL-10 such as *Borrelia burgdorferi and* murine* Leishmania *[14, 24].

In summary, we have demonstrated the ability of *C. trachomatis* MoPn Nigg II to induce the *in vitro* production of IL-6, IL-8, and TNF in macrophages and epithelial cells. We also show that IL-10 inhibits the production levels of these cytokines as elicited by live *C. trachomatis* in HeLa and macrophages, and by UV-inactivated *C. trachomatis* in macrophages. Our results clearly support our hypothesis that IL-10 may be an important regulator of inflammation during the initial stage of a *C. trachomatis* infection, when acquired immunity has not yet developed.
